# Comparison of body adiposity index and body mass index for assessment of body fat percentage among office workers: A validation study

**DOI:** 10.12669/pjms.41.6.11630

**Published:** 2025-06

**Authors:** Ramsha Habib, Syed Fawad Mashhadi, Ayesha Javed, Mommana Ali Rathore

**Affiliations:** 1 Ramsha Habib, MBBS, MPhil Department of Community Medicine/Public Health, Army Medical College, National University of Medical Sciences, Rawalpindi, Pakistan; 2 Syed Fawad Mashhadi, MBBS, MPH, MCPS, MPhil, Ph.D Professor/ Head of Department of Community Medicine/Public Health, Army Medical College, National University of Medical Sciences, Rawalpindi, Pakistan.; 3 Ayesha Javed, MBBS, MPhil, Department of Community Medicine/Public Health, Army Medical College, National University of Medical Sciences, Rawalpindi, Pakistan; 4 Mommana Ali Rathore, MBBS, PGD(nut), MPhil, CHPE, Department of Community Medicine/Public Health, Army Medical College, National University of Medical Sciences, Rawalpindi, Pakistan

**Keywords:** Body fat percentage, Body mass index, Body adiposity index, Bioimpedance analysis, Obesity, Office workers

## Abstract

**Objective:**

To determine the diagnostic accuracy of Body Mass Index (BMI) and Body Adiposity Index (BAI) in assessing body fat percentage among office workers.

**Methods:**

This validation study was carried out among office workers in WAPDA center offices of three districts (Abbottabad, Haripur and Mansehra) in Hazara division of Khyber Pakhtunkhwa province from July 2024 to December 2024.Two stage probability sampling was used to select the sample. Data were collected regarding sociodemographics, anthropometric measures, behavioural factors and body fat percentage was obtained through bioimpedance analysis. BMI and BAI were calculated and compared to body fat percentage derived from bioimpedance analysis (reference method).

**Results:**

Overall BAI showed better sensitivity of 81% (95%CI =70.6% - 88.9%) than BMI. ROC analysis showed greater AUC value of 0.85 (95% CI=0.79-0.91) in males and 0.97(95% CI=0.95-0.99) in females as compared to BMI (p < 0.05).Multiple linear regression showed age, weight and hip circumference to be positively associated with body fat percent (B values being 0.19, 0.28 and 0.33 respectively) while height, gender and physical activity were negatively associated with body fat percentage (B values -0.38,-0.43 and -0.26 respectively) (p<0.05).

**Conclusions:**

BAI is a better predictor of body fat as compared to BMI among office workers and can replace BMI as a screening tool to measure body fat in community settings.

## INTRODUCTION

Worldwide, obesity prevalence is sharply increasing, and it is currently considered the sixth most important risk factor for death worldwide and a serious public health concern.^1^ Since 1990, the global prevalence of adult obesity has more than doubled, while the rate of adolescent obesity has quadrupled. In 2022, one in eight persons worldwide suffered from obesity.^2^ The highest rates of obesity are seen in the American and European regions.^3^South Asian countries such as the Maldives, Bhutan, Myanmar, Nepal, and Bangladesh are among those experiencing the fastest increases in obesity. India and Pakistan are expected to have the highest rates of adult obesity by 2030.^4^Employment-related physical activity has decreased in recent years, which correlates with rising body weight leading to obesity and related health issues.^5^In a systematic review of studies investigating occupational characteristics related to obesity, 70% of studies indicated favorable relationships between lengthy work hours and weight-related outcomes.^6^

A sound and unquestionable estimate of body fat percentage (%BF) in the human body necessitates expensive diagnostics like dual-energy X-ray absorptiometry (DEXA) and Bioimpedance analysis (BIA) showing a high correlation with DEXA.^7^ The body mass index (BMI), a ratio between height and body weight, is the most commonly used anthropometric test to assess obesity in humans. However, BMI is limited in that it cannot distinguish between fat and lean body mass, and is affected by factors other than height and weight, such as age, gender, ethnicity, muscle mass, and activity level and as a result, its application may misclassify the weight status of athletic, physically active, and tall- and short-statured persons, whose lean-to-fat ratios and body proportions differ significantly from those of normal people.^8^ The Body Adiposity Index (BAI) was initially designed as a surrogate measure of adiposity and can be calculated as BAI = [Hip circumference (cm)/Height (m) ^1.5^] – 18.^9^ The measure was verified in African American adults before being used in the Mexican American community. In clinical settings, BAI is a rapid and cost-effective approach for determining body fat percentage.^10^

Comparison studies between BMI and BAI show different predictive capacities in different populations.^11^Although many comparison studies of BMI and BAI have been conducted in Pakistan but there is paucity of data about both of these indices being compared to a reference standard method. Also there is a significant shortage of data on these indices specifically within the office worker population. This study aimed to compare the effectiveness of the BAI and BMI as a screening tool in predicting body fat percentage among office workers with Bioimpedence analysis as a reference standard method, in order to determine which index provides a more accurate assessment of body fat percentage in this specific population.

## METHODS

This validation study was conducted among office workers in main WAPDA center offices of three districts (Abbottabad, Haripur and Mansehra) of Hazara Division Khyber Pakhtunkhwa. A meeting was held with the superintendent engineer of WAPDA offices to discuss the research purpose and obtain permission. Sample size of 288 was calculated through Cochrane formula keeping the confidence interval 95%, margin of error 0.05 and population proportion 0.25. The sample was drawn using two stage probability sampling technique. In the first stage, two out of three main WAPDA offices were selected through simple random sampling using computer generated numbers. In the second stage, stratified random sampling was used. Subjects in each selected office were stratified according to their gender (male or female) and sample was drawn from each stratum through simple random sampling. Equal allocation was used for sample selection from each stratum. This study was carried out from July 2024 to December 2024.Full time office workers aged 35 to 64 years employed at WAPDA offices were included in the study. Those excluded were part-time or temporary workers, pregnant women and workers with comorbidities or implanted electrical devices such as pacemakers. Sociodemographic information about age, gender, marital status, family history, education and monthly income was obtained. Physical activity and healthy eating habits were assessed using International Physical Activity questionnaire (IPAQ) scored as MET-min/week and Healthy Eating Assessment questionnaire (HEAQ) scored from 10 -50 based on identification of eating patterns over the past few weeks. Weight, height and hip circumference were recorded. Weight and height were measured using electronic scale (calibrated in kg) and stadiometer (m) respectively. Hip circumference (cm) was measured through a measuring tape at the level of maximum extension of buttocks. BMI was calculated as weight of the subject in kg divided by height in meters squared. Standard WHO BMI classification (cut off >30 kg/m^2^) was followed to maintain consistency with global standards and to allow comparison with BAI for which no population specific classification is available.

BAI was calculated as hip circumference / height^1.5^ – 18. Bioimpedance analysis was used as a reference standard method instead of DEXA because of its cost effectiveness and portability which makes it more suitable for field based research studies. Bioimipedance analysis was done through Omron Handheld body fat analyser HBF 306C to which the body fat percentage derived from BMI and BAI were compared in this study. Subjects were made to stand straight with both feet slightly apart, grab the device by electrodes with arms straight at 90° angle and thumbs on top of electrodes and readings were taken. Data was analysed using SPSS 27 and checked for normality by applying Kolmogorov-Smirnov (KS) test. Qualitative data (such as name, gender, marital status, employment status) was presented as percentage and frequencies. Quantitative data (such as age, weight, height, hip circumference, body fat %) was presented as mean and standard deviation. Sensitivity, specificity, positive predictive value and negative predictive value were derived. Receiver operator curves (ROC) were plotted and area under curves (AUC) determined to identify the discriminatory capacity of BMI and BAI for body fat percent (95% CI). Multiple regression models were used to assess the effect of different socio demographic variables and potential confounders (age, height, weight, gender, physical activity, healthy eating) on percent body fat. A p-value of < 0.05 was considered significant.

### Ethical Approval:

It was obtained from institutional ethical review committee of Army Medical College, NUMS (ERC/ID/413 – Dated: October 17, 2024).

## RESULTS

The present study included 288 (n=288) office workers out of which 144 were males and 144 were females. The mean age of the subjects was 42 ± 6 years. BMI ranged from 14.9 kg/m^2^ -49.2 kg/m^2^ (26.8 ± 5.6) while BAI ranged from 11.1% to 57.5% (30.7± 6.2). Other baseline and anthropometric measures are presented in Table-I.

**Table-I:** Baseline characteristics and anthropometric measures.

Variable	N	%
***Gender***		
Male	144	50
Female	144	50
***Marital status***		
Married	252	87.5
Unmarried	36	12.5
***Family History of CAD***		
Yes	112	38.9
No	176	61.1
***Family history of Hypertension***		
Yes	144	50
No	144	50
***Family history of Diabetes***		
Yes	130	45.1
No	158	54.9
***Family history of obesity***		
Yes	73	25.3
No	215	74.7
***Education level***		
Primary	13	4.5
Secondary	136	47.2
University level	139	48.3
***Monthly income***		
<50000	68	23.6
50000-100000	151	52.4
> 100000	69	24
	*Mean*	*SD*
Age(years)	42.2	6.2
Hip circumference(cm)	103.9	10
Height(m)	1.6	0.09
Weight(kg)	73	16.5
BMI(kg/m^2^)	26.8	5.6
BAI(%)	30.7	6.2
BimpA(%)	28.2	8.4

Table-II represents overall as well as gender specific sensitivity and specificity values for BMI and BAI according to body fat percentage derived through bio impedance analysis. At BMI cut-off value of > 30kg/m^2^, the overall sensitivity was 67% (95% CI 55.6% - 77.2%) and specificity 88% (95% CI 83.9 -92.8%).In comparison BAI had overall sensitivity of 81% (95% CI =70.6% - 88.9%) and specificity 82% (95% CI = 76.9% - 87.6%). After stratification by gender, BMI showed specificity of 91% (95% CI 83.7% - 96.2%) in males and 87% ( 95% CI 79.5% - 92.5%) while BAI showed sensitivity of 90% in males (95% CI= 78.5% - 96.7%) and specificity of 97% (95% CI=92.6% - 99.4%) in females. Total positive and negative predictive values as well as those for separate genders are also depicted in Table-II.

**Table-II:** Diagnostic accuracy of BMI and BAI.

	Males				Females				Total			
	Se	Sp	PPV	NPV	Se	Sp	PPV	NPV	Se	Sp	PPV	NPV
BMI	54%	91%	77%	78%	89%	87%	62%	97%	67%	88%	69%	87%
BAI	90%	64%	58%	92%	64%	97%	85%	91%	81%	82%	64%	92%

Se Sensitivity, Sp specificity, PPV positive predictive value, NPV negative predictive value

Overall, BAI showed higher NPV of 90% (95% CI 89.2% - 95.3%) whereas BMI showed higher PPV of 69% (95% CI= 57.2% - 75.4%). This indicates that BAI correctly identified 81% of obese subjects while BMI correctly classified only 67% of obese subjects. A receiver operator curve (ROC) analysis was carried out to evaluate the performance of BMI and BAI in predicting obesity using bio-impedance analysis as reference standard method. The area under the curve (AUC) was higher for BAI in both genders, 0.85(95% CI=0.79-0.91) in males and 0.97(95% CI=0.95-0.99) in females as compared to that of BMI which was 0.81(95% CI=0.74-0.88) and 0.93(95% CI=0.88-0.98) for males and females respectively. This showed that BAI has a higher discriminatory capacity for obese and non-obese as compared to BMI (Fig.1A and 1B).

**Fig.1A:**
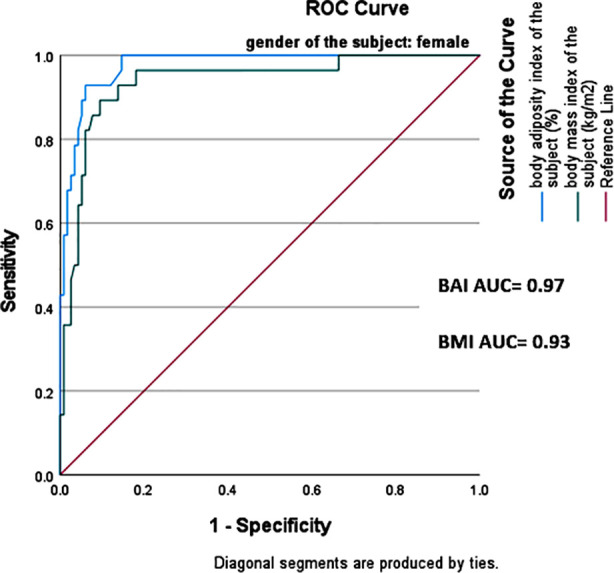
ROC curve for BMI and BAI in females.

**Fig.1B:**
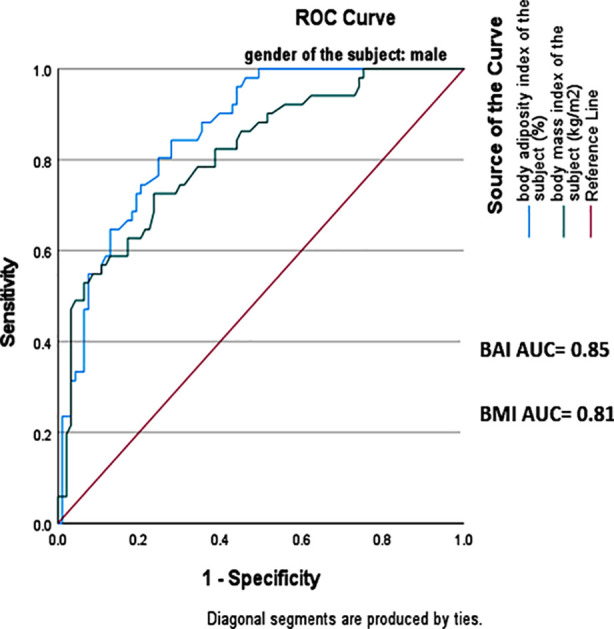
ROC curve for BMI and BAI in males.

A multiple linear regression analysis was performed to assess the relationship between body fat percentage and ten predictors. The overall regression was significant, R^2^ =0.66, p <0.001, suggesting that 66% of the variation is predicted by these factors. Specific predictors that influence the body fat percentage were age, height, weight, hip circumference, gender and physical activity (Table-III).The standardized coefficient beta (B) suggested that age, hip circumference and weight affected the body fat percentage positively, their B coefficient values being 0.19, 0.28 and 0.33 respectively (p<0.05).However, height, gender and physical activity score were negatively related to body fat percentage (B values -0.38,-0.43 and -0.26 respectively) (p<0.05).This indicates that with increasing age, weight and hip circumference values, the body fat percentage increases while opposite is true for height and physical activity. No significant association was observed between healthy eating habits and body fat percentage (B= 0.047, p = 0.190).

**Table-III:** Multiple linear regression for body fat %.

Variables	Unstandardized Coefficients		Standardized Coefficients Beta	T value	P Value
	B	St. Error			
Age	0.271	0.051	0.19	5.32	0.000*
Height	-34.846	5.113	-0.382	-6.815	0.000*
Weight	0.193	0.041	0.33	4.699	0.000*
Hip circumference	0.303	0.062	0.28	4.918	0.000*
Gender	-6.009	0.967	-0.43	-6.214	0.000*
Education Level	0.246	0.565	0.017	0.436	0.663
Monthly Income	-1.180	0.477	-0.96	-2.473	0.014
HEAQ score category	0.651	0.495	0.047	1.313	0.190
IPAQ score category	-5.5	0.93	-0.26	-5.9	<0.01*

* p value sig = <0.05.

## DISCUSSION

This study was carried out to compare BMI and BAI as measures of body fat with a reference standard method that is bioimpedance analysis among office workers. In this study sensitivity and specificity analysis of BMI and BAI were carried out. The results revealed, at cut-off value of 30kg/m^2^, BMI showed a poor sensitivity of 54% while a good specificity of 91%, similar to a study carried out in Saudi Arabia, the results of which showed 34% sensitivity and 98% specificity in men.^12^ Overall the sensitivity of BMI was found to be 67% in contrast to a study carried out in Brazil where it was very low (28%).^13^This difference might be attributed to different cut off values taken for BMI in the current study.

The sensitivity and specificity of BAI was determined to be 64% and 97% in females. A similar study carried out among females in Poland showed the sensitivity and specificity of BAI to be 41% and 94% respectively.^14^ BAI in men showed high sensitivity (90%) unlike another study of BAI done in India, where this value was 57%.^15^ ROC analyses was performed to identify AUC for BMI and BAI relative to body bioimpedance analysis. The AUC for BMI was 0.81 in males and 0.93 in females. These findings are comparable with a study done in USA which demonstrated similar results of AUC for BMI being higher in females than males.^16^

According to the results of this study, AUC for BAI was higher in females (0.97) as compared to males (0.85).This is in contradiction to the findings of a study carried out in Iran, where AUC curve for BAI in males was higher as compared to females.^17^However, according to a research conducted in Spain, AUC for BAI was of high value in females than that of males, which aligns with current study’s findings.^18^ The higher values of AUC curve for BAI in females may be attributed to the use of hip circumference in BAI equation to predict body fat and tendency of females to store more body fat in gluteal-femoral region while males tend to store more fat in abdominal region.

Multiple linear regression was applied to determine which predictors may explain variations in body fat percentage. According to results, age is positively related to body fat percentage which is consistent with findings from another study carried out by Macek et al in Poland.^19^ This might be explained by higher energy intake and reduced mobility as people age. Physical activity was negatively associated with body fat percentage (B= -0.26, p <0.05) aligning with the results of a study carried out in China, where inverse relation between physical activity and body fat percentage was found.^20^ No significant association was found between healthy eating and body fat percentage(B= 0.047, p = 0.190).In contrast, results of a similar study carried out in the USA showed a weak association between body composition and diet quality.^21^This lack of significant association may be attributed to the questionnaire used for eating assessment (HEAQ) which relies on self-reported dietary data leading to recall bias and reduced accuracy.

This study adds to the limited evidence on the accuracy of BAI and BMI in estimating body fat among office workers of Pakistan using bioimpedance analysis as a reference. Its findings are clinically relevant as they inform the selection of practical, cost-effective screening tools for identifying individuals at risk of excess body fat in workplace settings, especially in resource-constrained environments.

### Strength of the study:

A key strength of this study is both BMI and BAI being compared to a standard reference method, strengthening the reliability of results. Another key strength of this study is relevance of the population that was chosen. Office workers are often characterized by sedentary lifestyles, long working hours and unhealthy eating habits making them prone to obesity and related outcomes. This study achieved a good response rate enhancing the reliability of the results. Lastly, the use of robust statistical techniques has provided a comprehensive evaluation of the diagnostic performance of both the indices. Future studies should explore how well BMI and BAI perform across various demographic groups other than office workers. Studies comparing both of the indices (BMI and BAI) with the gold standard method DEXA are also needed to validate their true effectiveness. Additionally, the impact of various factors such as alcohol consumption, medication use, smoking and metabolic conditions on body fat composition should also be investigated for enhanced applicability of these indices across diverse populations.

### Limitations

Despite yielding interesting results, this study is subject to various limitations. First of all, the study population (office workers) may not be representative of the entire population of the said province. Secondly, the reference standard method used in this study cannot be used in certain conditions such as edema, pregnancy and implanted electrical devices because these conditions can distort the results. Measurement bias was also a potential limitation of this study in recording of anthropometric measures. It was addressed by using standardized techniques and calibrated instruments. Measurements were taken thrice and average was obtained. Although physical activity and healthy eating habits were addressed in the study but other factors such as smoking, alcohol consumption, metabolic conditions, and medication use were not considered in analysis and may have contributed to variability in body fat composition among participants.

## CONCLUSION

There is a difference in performance of BAI and BMI in predicting body fat percentage when compared to a reference standard method (Bioimpedance Analysis). BAI performed better than BMI in terms of predicting body fat indicating its potential as a more reliable and practical screening tool for obesity. Findings can help governments drive public health policy and improve workplace wellness programmed by determining the optimum method for measuring body fat leading to better employee health.

### Disclaimer

It is hereby declared that this research article is our own autonomous work. All sources used have been indicated as such. All texts either quoted directly or paraphrased have been indicated by in-text citations. Full bibliographic details are given in the reference list.

### Authors’ Contribution:

**RH:** Conception, design, data collection, analysis, manuscript draft, final approval of manuscript, critical revision. She is also responsible for the accuracy of the study.

**SFM:** Conception, design, review at all stages, final approval of manuscript, accountability.

**AJ and MAR:** Contributed to literature search and critically reviewed the article.

All authors have approved the final version of the manuscript.
